# Efficacy and safety of sacituzumab govitecan in patients with metastatic metaplastic triple-negative breast cancer: a multinational retrospective case series from CEBCC-102 study

**DOI:** 10.1007/s10549-025-07875-4

**Published:** 2025-12-09

**Authors:** Justyna Żubrowska, Małgorzata Pieniążek, Anna Polakiewicz-Gilowska, Miroslava Malejčíková, Miloš Holánek, Renáta Soumarová, Aleksandra Konieczna, Iwona Danielewicz, Maja Lisik-Habib, Aleksandra Łacko, Marcin Kubeczko, Renata Pacholczak-Madej, Zuzana Bielčiková, Mirosława Püsküllüoğlu

**Affiliations:** 1 Department of Clinical Oncology, Holy Cross Cancer Center, Kielce, Poland; 2 Department of Oncology, Breast Unit Clinical Oncology Day Care Department, Lower Silesian Comprehensive Cancer Center, Wrocław Medical University, Wrocław, Poland; 3 Breast Cancer Center, Maria Skłodowska-Curie National Research Institute of Oncology, Gliwice, Poland; 4 Oncology Clinic of LFUK, National Cancer Institute, Bratislava, Slovakia; 5 Department of Comprehensive Cancer Care, Faculty of Medicine, Masaryk University, Masaryk Memorial Cancer Institute, Brno, Czech Republic; 6 Department of Oncology, Third Faculty of Medicine, Charles University, University Hospital Královské Vinohrady (FNKV), Prague, Czech Republic; 7 Department of Breast Cancer and Reconstructive Surgery, Maria Skłodowska-Curie National Research Institute of Oncology, Warsaw, Poland; 8 Department of Oncology and Radiotherapy, Szpitale Pomorskie Sp. Z O.O, Gdynia, Poland; 9 Department of Proliferative Diseases, Nicolaus Copernicus Multidisciplinary Centre for Oncology and Traumatology, Łódź, Poland; 10 Department of Gynecological Oncology, Maria Skłodowska-Curie National Research Institute of Oncology, Kraków, Poland; 11 Department of Oncology, First Faculty of Medicine, Charles University and General University Hospital, Prague, Czech Republic; 12 Department of Clinical Oncology, Maria Skłodowska-Curie National Research Institute of Oncology, Kraków, Poland; 13 Department of Anatomy, Jagiellonian University Medical College, Kraków, Poland

**Keywords:** Sacituzumab govitecan, Antibody–drug conjugate, Triple-negative breast cancer, Metaplastic breast cancer, Real-world evidence

## Abstract

**Background:**

Metastatic metaplastic triple-negative breast cancer (mMpTNBC) is a rare, aggressive subtype with poor responsiveness to standard therapies. Sacituzumab govitecan (SG) is effective in metastatic TNBC (mTNBC), but data in mMpTNBC are limited to case reports. Patients and methods: This multinational, multicenter, retrospective case series was conducted within the CEBCC-102 real-world evidence project across 18 cancer centers. Female patients with histologically confirmed mMpTNBC treated with ≥ 2L SG outside of clinical trials in Poland, the Czech Republic and Slovakia between August 2021 and June 2025 were included. Clinical data, treatment outcomes, and adverse events (AEs) were collected from medical records and analysed.

**Results:**

Among 303 patients with mTNBC treated with SG in second and later lines within the CEBCC-102 project, 13 women (4.3%) had mMpTNBC and were included in this analysis. Median age was 58 years. PD-L1 CPS ≥ 10 was found in 83% of tested cases. Overall response rate was 36.4%, clinical benefit rate 45.5%, median progression-free survival 3.2 months and median overall survival 8.9 months. Neutropenia (N = 9, 69%) was the most common AE; no febrile neutropenia or treatment discontinuations due to toxicity occurred.

**Conclusions:**

This first international real-world series of SG in mMpTNBC shows clinically relevant activity and manageable toxicity, addressing a critical evidence gap and supporting further prospective studies, particularly in PD-L1–positive disease.

## Introduction

Metaplastic breast cancer (MpBC) is a rare, aggressive subtype accounting for approximately 0.2–5% of all breast cancers (BC) and characterized by heterogeneous histological features [1–3]. This entity encompasses poorly differentiated tumors with diverse morphological features that combine epithelial and mesenchymal elements, including spindle-cell morphology, squamous differentiation and chondroid or osseous components. Histological variants of MpBC include carcinosarcoma, matrix-producing carcinoma, sarcomatoid carcinoma, pseudosarcoma and mixed metaplastic tumors of the breast [1–4]. It typically demonstrates triple-negative receptor status, meaning tumors lack estrogen receptor (ER), progesterone receptor (PR) and human epidermal growth factor receptor 2 (HER2) overexpression or amplification [1–3]. Metastatic metaplastic triple-negative breast cancer (mMpTNBC) carries a particularly poor prognosis, reflecting its aggressive biology and low sensitivity to conventional systemic therapies [5, 6]. Due to its rarity, patients with mMpBC are largely underrepresented in prospective randomized trials. Therapeutic decisions are generally extrapolated from treatment principles established for other TNBC subtypes [7, 8].

Sacituzumab govitecan (SG), an antibody–drug conjugate (ADC), has demonstrated significant clinical efficacy in pretreated metastatic TNBC patients. In the phase III ASCENT trial, SG significantly prolonged median progression-free survival (PFS; 5.6 vs. 1.7 months, HR 0.41) and overall survival (OS; 12.1 vs. 6.7 months, HR 0.48) compared with physician's choice of chemotherapy, establishing a new standard in later-line metastatic TNBC treatment [9]. Available real-world evidence (RWE) supports the clinical benefit of SG in routine practice across numerous populations [10–13]. Patients with MpBC were underrepresented or absent in these studies.

The aim of this study was to assess the efficacy and safety of SG in mMpTNBC within a predefined subgroup of a multinational real-world study of second- or later-line metastatic TNBC.

## Methods

### Study design and study population

This study was conducted as a multinational, multicenter, retrospective case series within the framework of the Central European Breast Cancer Collaboration (CEBCC)−102 RWE project regarding patients with mTNBC treated with SG in second and later lines. Clinical data were collected from patients with mMpTNBC treated with SG outside of clinical trials across 18 oncology centers in Poland, the Czech Republic and Slovakia between August 2021 and June 2025. The case series included adult patients (≥ 18 years). Diagnosis was established through pathology review according to the World Health Organization (WHO) classification criteria. All patients had radiologically documented metastatic disease and had received at least one prior systemic therapy for advanced BC. Eligibility was irrespective of Eastern Cooperative Oncology Group** (**ECOG) performance status (PS) and comorbidities, provided that adequate clinical documentation was available. Patients with second active malignancies were excluded. Given the predefined case-series objective and rarity of mMpTNBC, all analyses were descriptive and no comparative statistics were performed.

### Data collection and standardization

Clinical data were retrospectively collected from medical records, including demographic information, disease-specific clinical parameters, prior systemic therapies, metastatic sites, ECOG PS, and treatment details, including dose modifications. Efficacy and safety assessments were conducted by experienced investigators.

### Treatment regimen

All patients received SG intravenously at a dose of 10 mg/kg on days 1 and 8 of a 21 day cycle, in accordance with the approved prescribing information. Dose delays and modifications were applied at the discretion of the treating physician in response to treatment-emergent toxicities, following the summary of product characteristics (SmPC), institutional standards, and local reimbursement requirements.

### Outcome measures

The primary efficacy endpoints included the overall response rate (ORR), defined as the proportion of patients achieving either a complete response (CR) or partial response (PR) as their best overall response, and the clinical benefit rate (CBR), defined as the proportion of patients whose best overall response was CR, PR, or stable disease (SD) maintained for a duration of at least 6 months (as per the ASCENT trial), assessed according to the Response Evaluation Criteria in Solid Tumors (RECIST) version 1.1, as well as median PFS and OS. PFS was defined as the time from SG initiation to the PD or death from any cause, whichever occurred first. OS was defined as the time from SG initiation to death from any cause. Safety was assessed as the incidence and severity of treatment-emergent adverse events (AEs), graded according to the Common Terminology Criteria for Adverse Events (CTCAE) version 5.0 and occurring from the start of SG up to 30 days after the last dose. Given the retrospective design and limited patient numbers, safety outcomes were summarized descriptively. Secondary endpoints included the frequency of treatment modifications (dose reductions, delays, or discontinuations due to toxicity). Baseline cohort characterization comprised programmed death-ligand 1 (PD-L1) expression, combined positive score (CPS), *breast cancer susceptibility* (*BRCA*) mutation status, and sites of metastasis.

### Statistical analysis

Descriptive statistics were employed to summarize baseline patient and disease characteristics. Continuous variables were presented as median values and categorical variables were summarized as frequencies and percentages. Survival outcomes (PFS, OS) were estimated using Kaplan–Meier method, with median values and 95% confidence intervals (CIs) reported. Statistical analyses were conducted using statistical software packages (e.g., R version 4.3, R Foundation for Statistical Computing, Vienna, Austria). Statistical significance was defined at a p-value < 0.05.

### Ethical considerations

The study protocol was approved by the ethics committees of the Maria Sklodowska-Curie National Research Institute of Oncology in Krakow (2/2023, 18 April 2023) and Warsaw (21/2024, 22 February 2024), as well as by the ethics committee of the Masaryk Memorial Cancer Institute in Brno (1737/2025, 10 June 2025). It was conducted in accordance with local regulations, Good Clinical Practice guidelines, and the Declaration of Helsinki (1964) and its subsequent amendments. Patient confidentiality and data protection were maintained according to the General Data Protection Regulation and local privacy laws. Given the retrospective design, the requirement for informed consent was waived by the ethics committees.

## Results

### Patients

Thirteen female patients treated in eight cancer centers met the eligibility criteria for this analysis, accounting for 4.3% of the metastatic TNBC study population. At the time of SG initiation, the median age was 58 years (range 31–73). The median number of prior lines of palliative treatment before SG was 2 (range 1–3). PD-L1 status was available for six patients, of whom five had CPS ≥ 10. *BRCA* mutation testing was performed in 10 patients, and no pathogenic variants were identified in any of them. Metastatic spread most commonly involved the lungs (76.9%). Only one patient had brain metastases at the time of treatment initiation. The median number of metastatic sites was 3 (range 1–5). A comprehensive summary of patient and disease characteristics, as well as treatment outcomes, is provided in Table 1.

**Table 1:** Patient and disease characteristics and treatment outcomes

Parameter	Patients, N (%)
Primary treatment intention	
Palliative	3 (23.1%)
Radical	10 (76.9%)
History of ER- or PgR-positive BC	
No	12 (92.3%)
Yes	1 (7.7%)
History of HER2-positive BC	
No	13 (100%)
Yes	0 (0%)
*BRCA1* or *BRCA2* mutational status	
Negative	10 (76.9%)
Positive	0 (0%)
Unknown	3 (23.1%)
PD-L1 CPS ≥ 10	
No	1 (7.7%)
Yes	5 (38.5%)
Unknown	2 (15.4%)
Not performed	5 (38.5%)
ECOG performance status	
0	5 (38.5%)
1	8 (61.5%)
2	0 (0%)
> 2	0 (0%)
Comorbidities	
No	8 (61.5%)
Yes	5 (38.5%)
Menopausal status	
Premenopausal	3 (23.1%)
Postmenopausal	10 (76.9%)
Site of metastatic disease	
Lymph nodes	8 (61.5%)
Lung	10 (76.9%)
Bones	2 (15.4%)
Liver	4 (30.8%)
Skin/subcutaneous tissue	3 (23.1%)
Malignant effusion	3 (23.1%)
Brain	1 (7.7%)
Other	2 (15.4%)
Number of measurable lesions	
1–3	5 (38.5%)
4–10	4 (30.8%)
10–20	2 (15.4%)
Best radiological response	
CR	0 (0%)
PR	4 (30.8%)
SD	5 (38.5%)
PD	2 (15.4%)
Unknown	2 (15.4%)
Progression	
No	4 (30.8%)
Yes	7 (53.8%)
No data	2 (15.4%)
Type of progression	
New lesion	3 (23.1%)
Progression of target lesions	4 (30.8%)
Clinical progression	0 (0%)
Unknown	0 (0%)
Death	
No	5 (38.5%)
Yes	8 (61.5%)

*BC* breast cancer, *BRCA* breast cancer susceptibility gene, *CPS* combined positive score, *CR* complete response, *ECOG* Eastern cooperative oncology group, *ER* estrogen receptor, *HER2* human epidermal growth factor receptor 2, *PD* progressive disease, *PD-L1* programmed death-ligand 1, *PgR* progesterone receptor, *SD* stable disease

### Efficacy outcomes

**Fig. 1:**
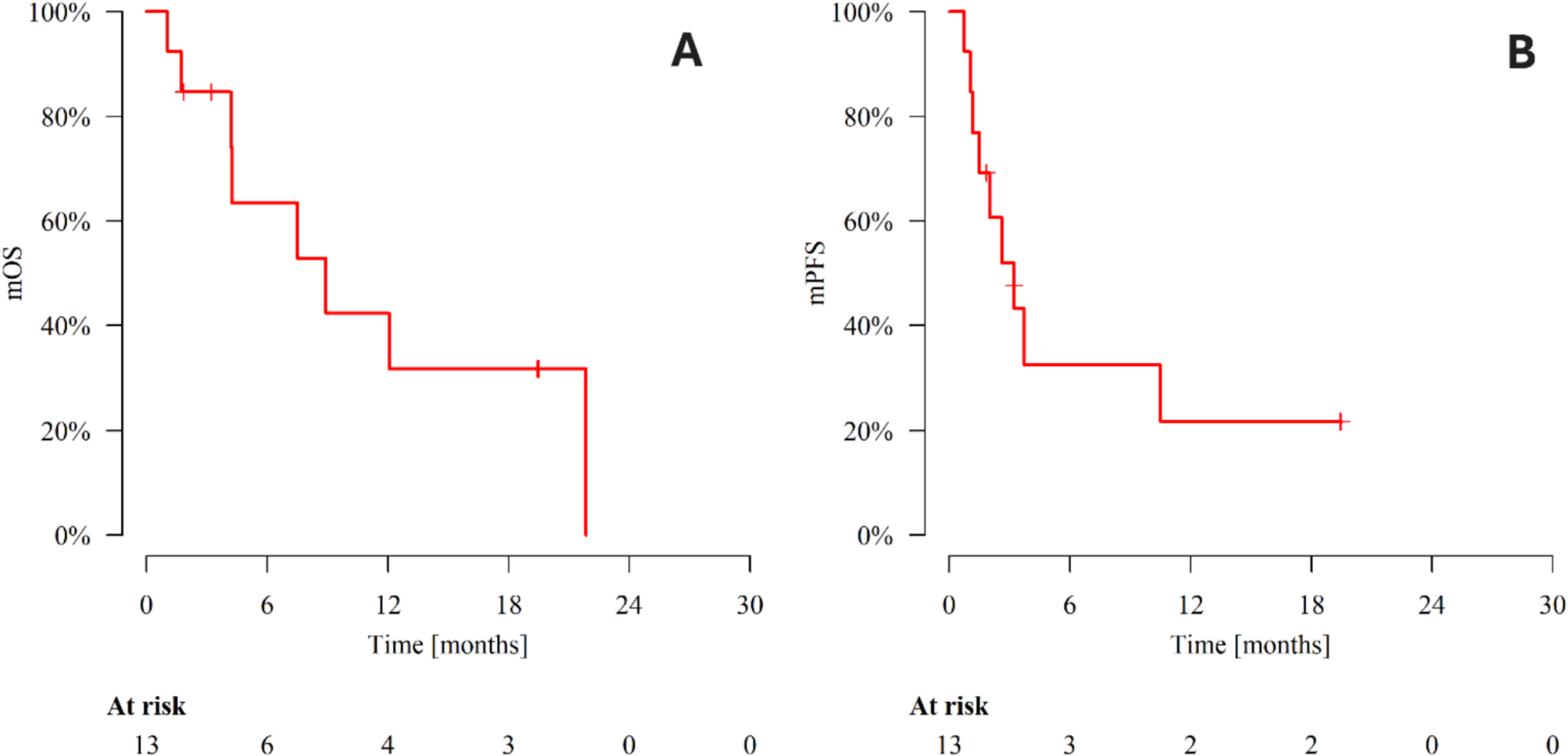
Kaplan–Meier curves for overall survival **A** and progression-free survival **B**. *mOS* median overall survival, *mPFS* median progression-free survival

The median follow-up was 4.2 months (range 1.1–21.9). At the time of analysis, eight patients had died and SG was discontinued in nine patients. The most common reason for treatment discontinuation was disease progression, reported in seven patients (77.8%). In patients with response assessed per RECIST v1.1 (n = 11), the ORR was 36.4%, while the CBR was 45.5%. Three patients who had progression on SG initiated subsequent systemic treatment. In this case series, median OS was 8.9 months and median PFS was 3.2 months. OS and PFS are presented in Table 2 and Fig. 1.

**Table 2:** Progression-free survival and overall survival data

Outcomes	Patients	Events	3 months	6 months	9 months	12 months	Median [months]
Progression-free survival	13	9	51.9%	32.5%	32.5%	21.6%	3.2
Overall survival	13	8	84.6%	63.5%	42.3%	42.3%	8.9

### Safety outcomes

Nine patients (69.2%) experienced treatment delays at some point due to AEs, with a median time to the first delay of 28 days (range 8–85). The most frequent cause of first treatment delay was neutropenia (N = 6, 46.1%). Five patients (38.5%) required dose reduction, with median time to the first dose reduction of 44 days (range 8–85). The most common cause for dose reduction was also neutropenia (N = 4, 30.8%). None of the patients discontinued SG treatment due to AEs. Most AEs were mild to moderate in severity, with only a few grade 3–4 toxicities reported. A detailed summary of AEs is presented in Table 3**.**

**Table 3:** Summary of adverse events

Type of adverse event	Patients, N (%)	Grade 1–2, N (%)	Grade 3–4, N (%)	Unknown, N (%)
Anemia	6 (46.1)	3 (23.1)	3 (23.1)	–
Thrombocytopenia	1 (7.7)	–	1 (7.7)	–
Neutropenia	9 (69.2)	5 (38.5)	4 (30.8)	–
Febrile neutropenia	0 (0)	–	–	–
Diarrhea	3 (23.1)	3 (23.1)	–	–
Nausea	6 (46.1)	5 (38.5)	–	1 (7.7)
Vomiting	2 (15.4)	2 (15.4)	–	–
Hepatic toxicity	1 (7.7)	1 (7.7)	–	–
Hypersensitivity reactions	1 (7.7)	–	1 (7.7)	–
Fatigue	5 (38.5)	5 (38.5)	–	–

## Discussion

In this first multinational, multicenter retrospective case series, SG demonstrated clinically relevant activity in patients with mMpTNBC. The overall efficacy was modest, with a median PFS of 3.2 months and OS of 8.9 months, and a clinical benefit observed in approximately half of the patients, comparable to outcomes reported in clinical trials. The treatment was generally well tolerated.

The efficacy and manageable safety profile of SG were established in the phase III ASCENT trial [9]. Although the ASCENT trial demonstrated consistent benefit across all predefined clinical subgroups—including patients previously treated with PD-1/PD-L1 inhibitors and those with brain metastases—it enrolled a heterogeneous TNBC population and did not include subgroup analyses according to histopathological subtype. Histopathological stratification has likewise not been assessed in available RWE [11–13], leaving the activity of SG in mMpTNBC largely undefined. Consequently, data specific to this subgroup remain extremely limited. The only broader source is a retrospective study by Maheswaran et al., reported exclusively in a conference abstract, which included both MpTNBC and non-MpTNBC, as well as patients treated within clinical trials and with combination regimens [14]. In that study, patients with MpTNBC treated with SG demonstrated a numerically shorter median PFS compared with those with non-MpTNBC, while OS was comparable between the groups. Beyond that, published evidence is limited to isolated case reports of MpBC [15, 16]. To our knowledge, this is the first international real-world series that substantially expands SG monotherapy activity in mMpTNBC.

The prevalence of mMpTNBC in our series was 4.3%. This finding is consistent with published data [3, 17, 18]. Direct comparison of baseline characteristics with the ASCENT trial is limited by the small sample size of our case series and incomplete clinical information. In our cohort, the lungs (77%) and lymph nodes (62%) were the most frequently involved metastatic sites, followed by the liver (31%). The ASCENT trial reported a similar frequency of liver (32%) and bone (22%) metastases. This may indicate differences in dissemination patterns between the two populations. The high rate of pulmonary involvement in our series aligns with prior reports, supporting the tendency of MpBC to metastasize to the lungs more often than other TNBC subtypes [3, 19].

In the presented analysis, the ORR was 36.4% and the CBR 45.5%, comparable to the phase III ASCENT trial, where these rates in the TNBC population were 35% and 45.6%, respectively. No CR were observed. However, one published case report described the CR to SG in a patient with mMpTNBC [15], highlighting the potential for deep responses in selected cases. Median OS and PFS were both shorter than reported in ASCENT, but only slightly lower than those reported in other RWE studies, where median OS typically ranged from 10 to 13 months and median PFS from 4 to 6 months [11–13]. Compared with the previously mentioned Maheswaran et al. cohort, PFS was longer, but OS was shorter [14]. In that analysis, mMpTNBC appeared less responsive to SG, yet patients achieved longer OS, possibly reflecting benefit from subsequent treatment lines or differences in treatment sequencing.

Despite the small cohort size, the safety profile of SG was consistent with the predictable and manageable toxicity reported in the ASCENT trial and RWE studies [11–13]. Most AEs were grade 1–2, while grade 3–4 events were infrequent and did not result in treatment discontinuation. Neutropenia was the most common AE. However, no cases of febrile neutropenia occurred. Anemia was observed in six patients (46.1%), with grade 3–4 severity in three (23.1%), a higher and somewhat atypical incidence compared with 8% in ASCENT and 1.7–10.1% in RWE reports [11–13]. Other AEs were typical for SG and included mild gastrointestinal symptoms and fatigue.

In our series, PD-L1 status was assessed in six patients, five of whom (83.3%) had a CPS ≥ 10. This proportion was higher than typically reported in TNBC and is consistent with previous reports of frequent PD-L1 expression in MpBC [20], supporting the potential role of immune checkpoint inhibitors or combination strategies. Germline *BRCA1/2* mutation testing was performed in ten patients with no pathogenic variants detected, despite reports of higher mutation prevalence in MpBC compared with other TNBC subtypes [21]. The absence of such alterations limits the applicability of PARP inhibitors. Nevertheless, genetic testing remains important, and broad genomic profiling may reveal additional actionable targets relevant to MpBC management.

This study has several limitations. First, its retrospective design carries the risk of selection bias, incomplete documentation, and heterogeneity in data collection across centers. Second, the small sample size, reflecting the rarity of mMpTNBC, limits the statistical power and generalizability of the findings. Time-to-event outcomes (PFS, OS) and response rates according to RECIST v1.1 should be regarded as exploratory. We did not include a contemporaneous comparator cohort of non-metaplastic mTNBC treated with SG. Such an analysis was not within the scope of this case-series report. However, RWE from the broader mTNBC population in this part of Europe is available and these data confirm the effectiveness and safety of SG in routine clinical practice but do not provide information specific to the mMpTNBC subgroup [13, 22]. Furthermore, histological subtyping of metaplastic carcinoma was not uniformly available across participating centers and could not be reliably abstracted. The absence of subtype-level reporting represents an additional limitation given the biological heterogeneity of this entity and should be addressed in future prospective studies incorporating centralized pathology review. Variations in clinical management, including supportive care and reimbursement policies, may have influenced treatment delivery and AE management. Finally, the absence of a comparator group precludes direct evaluation of SG against alternative therapeutic strategies.

## Conclusion

Despite these limitations, this case series provides valuable RWE on the use of SG in a subgroup of patients who remain substantially underrepresented in both prospective clinical trials and available real-world analyses. The observed outcomes offer an early indication that SG may retain clinically meaningful activity regardless of histological subtype, thereby addressing an important gap in the current evidence base. Our report expands upon the efficacy and safety signals established in the ASCENT trial and complements existing RWE describing the use of SG in TNBC populations. Given the rarity and biological heterogeneity of MpBC, this entity continues to pose a significant therapeutic challenge. These findings underscore the need for dedicated clinical studies and the ongoing development of novel therapeutic strategies for this difficult-to-treat subtype.

## Data Availability

No datasets were generated or analysed during the current study.
